# Identification and utilization of a new *Bacillus amyloliquefaciens* XY-1 against Fusarium head blight

**DOI:** 10.3389/fpls.2022.1055213

**Published:** 2022-11-30

**Authors:** Xiao Xu, Yifan Cheng, Zhengwu Fang, Junliang Yin, Huiquan Shen, Dongfang Ma

**Affiliations:** ^1^ Key Laboratory of Sustainable Crop Production in the Middle Reaches of the Yangtze River (Co-construction by Ministry and Province), College of Agriculture, Yangtze University, Jingzhou, China; ^2^ Jiangsu Academy of Agricultural Sciences, Jiangsu Coastal Area Institute of Agricultural Sciences, Yancheng, China

**Keywords:** *Bacillus amyloliquefaciens*, Biological control, Fusarium head blight, *Antagonistic bacterium*, XY-1

## Abstract

Fusarium head blight (FHB) is a global wheat grain disease caused by *Fusarium graminearum*. Biological control of FHB is considered to be an alternative disease management strategy that is environmentally benign, durable, and compatible with other control measures. In this study, to screen antagonistic bacteria with the potential to against FHB, 45 strains were isolated from different tissues of wheat. Among them, seven strains appeared to effectively inhibit *F. graminearum* growth, the antagonistic bacterium named XY-1 showed a highly antagonistic effect against FHB using dual culture assays. The strain XY-1 was identified as Bacillus amyloliquefaciens by 16S rDNA sequence. Antibiotic tolerance of antagonistic bacteria showed that XY-1 had antagonistic activity against Colletotrichum gloeosporioides, Rhizoctonia solani, Sclerotium rolfsii, and Alternaria alternata. Nutrition tests showed that the most suitable carbon and nitrogen sources were glucose and beef extract, respectively. The optimum growth temperature and pH value were 28 ℃ and 7.4. Antibiotics tolerance cultivation showed that XY-1 had strong resistance to Chloramphenicol and Ampicillin. Wheat spikes inoculation antagonism tests showed that strain XY-1 displayed strong antifungal activity against *F. graminearum*. Our study laid a theoretical foundation for the application of strain XY-1 as a biological agent in the field to control FHB.

## Introduction

Fusarium head blight (FHB) is a global wheat grain disease caused by *Fusarium graminearum* (Senatore et al., 2021). The incidence of FHB increases with the increase in the area under wheat cultivation (Liu et al., 2016; Li et al., 2017). Moreover, the byproduct (deoxynivalenol (DON)) of wheat infected by *F. graminearum* can cause human and livestock poisoning (Li et al., 2022). As a consequence, wheat grains with severe head blight infection cannot be processed into flour or feed, resulting in significant economic losses (Miedaner et al., 2003; William et al., 2004; Ma et al., 2015). FHB is prevalent in the winter wheat area of the Yangtze River Basin and in areas with relatively high humidity, yield loss can reach up to 80% in some wheat fields (Zhang et al., 2014). Chemical control is still the best control method against *F. graminearum*. However, there is growing concern about the negative effects of chemical pesticides, particularly about their potentially toxic effects on humans and animals (Crane et al., 2013). Thus, alternative disease management strategies are needed to fulfill the consumer demand for pesticide-free food while maintaining environmental safety (Lamichhane et al., 2016). The use of biological control is being considered as a good alternative in the prevention and control of plant diseases (Rahman et al., 2018).

Plants provide good habitats for endophytes, and they have a wide range of beneficial biological control functions for host plants. Existing studies had shown that endophytes affect host plants through their own metabolites and signal transduction. Endophytic bacteria could secrete peptide (lipopeptide), fat gambogic pyocyanin (pyoluterin), pyrrole cephalosporins (pyrolnit rin), as well as chitinase and glucanase such as antibacterial materials (Sun et al., 2006). These antibacterial substances could degrade bacterial mycelia or pathogenic factors and had good antibacterial effect on plant pathogens. *Trichoderma afroharzianum*, evaluated for the first time in Algeria as biocontrol agent, is a promising biocontrol approach against FCR and FHB (Bouanaka et al., 2021).

*Bacillus amyloliquefaciens* has been widely identified as a biological control microbe against *F. graminearum*. *Bacillus amyloliquefaciens* FZB42 is a Gram-positive plant growth-promoting bacterium with an impressive capacity to synthesize nonribosomal secondary metabolites with fungal activity (Hanif et al., 2019). The commercially available strain *B. amyloliquefaciens* FZB42 showed strong activity against *F. graminearum* and the lipopeptide *bacillomycin* D, produced by FZB42, was shown to contribute to the antifungal activity (Gu et al., 2017). A bacterial strain (S76-3, identified as *Bacillus amyloliquefaciens*) that was isolated from diseased wheat spikes in the field displayed strong antifungal activity against *F. graminearum* (Gong et al., 2015). *B. amyloliquefaciens* JCK-12 could be used as an available biocontrol agent or as a chemosensitizer to chemical fungicides for controlling FHB disease and as a strategy for preventing the contamination of harvested crops with mycotoxins (Kim et al., 2017). The lower iturin levels on wheat spikes in the field could be a major factor limiting disease control in field settings (Crane et al., 2013). In this study, endophytic bacterium named XY-1 was isolated from healthy wheat plants collected from areas with severe wheat head blight. After the optimization of culture conditions, physiological and biochemical identification, resistance examination and 16S rDNA sequence analysis were carried out to determine their antibiotic tolerance and biological species. The resulting data provided a theoretical basis for an alternative method of biological control of FHB.

## Materials and methods

### Pathogenic fungal strains

*Fusarium graminearum* was provided by the Institute of Plant Protection, Jiangsu Academy of Agricultural Sciences. *Colletotrichum gloeosporioides* (an apple fruit pathogen), *Rhizoctonia solani* (a cotton pathogen), *Sclerotium rolfsii* (a rice pathogen), and *Alternaria alternata* (a pear fruit pathogen) were acquired from the lab of Pathogenic Fungi and Genomics in College of Agriculture, Yangtze University, Jingzhou, China.

### Isolation and purification of endophytic bacteria

The samples were collected by five-point sampling method in Jingzhou, Hubei Province from plants grown in a field with severe wheat scab outbreak (N30°21′32.53″ E112°08′22.05″). The roots, stems, leaves, and spikelets of wheat were cut into small segments of about 5 cm and washed with water for 30 min. The following operations were carried out on a clean bench: samples were washed 4 times with sterile water, soaked in 75% ethanol for 5 min, washed with sterile water two times, soaked in 1% mercuric chloride for 3 min, and finally rinsed three times with sterile water. The sterilized plant material was placed in a sterile mortar containing an appropriate amount of quartz sand. The sample was grinded with 10 ml of sterile water, 100 μL of the solution was plated on Potato Dextrose Agar (PDA: potato 20 g/L, glucose 20 g/L, agar 15 g/L) and Nutrient Agar (NA: peptone 10 g/L, beef extract 3 g/L, glucose 2.5 g/L, agar 18 g/L) kept at a constant temperature of 28°C After three days of culture, colonies were separated and cultured on PDA and NA media, respectively. Pure strains were obtained after three times’ sub-culturing.

### Dual culture assays of antagonistic strain

Adopting dual culture assays tested the antagonistic effects of isolations on *Fusarium graminearum* (Su et al., 2010). In each repetition, a 5 mm mycelium pellet of *F. graminearum* was inoculated in the central portion of the PDA medium with a diameter of 90 mm, and the endophyte was attached to the periphery. Plates grown with *F. graminearum* alone served as controls, and three repetitions were set for each treatment. These were cultured at 28°C in the dark for 3 to 4 days until control dishes were covered with the *F. graminearum.* The inhibition zone was measured and statistically analyzed. The diameter of the zone of inhibition was the growth width of the antagonistic colonies, measured in a criss-cross method. Diameter of inhibition zone is calculated using the following formula: (A1+A2)/2, where A1 and A2 are respectively the diameter of inhibition zone measured by the criss-cross method. Two independent experimental replicates were performed for each strain. Dual culture assays were used to detect the inhibitory effect of antagonistic endophyte on the previous pathogenic fungi. Three replicates were performed for each treatment.

### Classification of endophyte isolates

DNA of the antagonize endophytes were extracted by using CTAB method (Liu et al., 2019). The extracted DNA was used as a template for polymerase chain reaction (PCR) to amplify with the 16S rDNA universal primers (27F: 5’-CAGAGTTTGATCCTGGCT-3’, 1492R: 5’-AGGAGGTGATCCAGCCGCA-3’) (Redburn and Patel, 1993). Amplification process order for: 94°C 3 min, 94°C 40 s, 65°C 40 s, 72°C 1 min, a total of 30 cycles; then 72°C 8 min. The amplified product was sequenced by Sangon Biotech. The sequencing results were used to perform BLAST searching on the NCBI website (http://www.ncbi.nlm.nih.gov/) to determine the species level. Phylogenetic tree was completed by the phylogenetic software MEGA 7.0 (Yin et al., 2017).

### Antibiotic tolerance of antagonistic bacteria

Five common antibiotics, *chloramphenicol* (Ch1)*, ampicillin* (Amp)*, tetracycline* (Tet)*, streptomycin* (Str) and *kanamycin* (Kan), were used to test the tolerance of strain XY-1. Bacterial suspensions from the colonies developed in NB were standardized to 108 CFU/mL and transferred (10 μL) to NA plates containing different concentrations of antibiotics (25 μg/mL CH1, 100 μg/mL Amp, 25 μg/mL Tet, 50 μg/mL Str, 10 μg/mL Kan).The plates were incubated at 28°C for 48 hand evaluated for the presence of bacterial growth. The colony diameter of 0-8 mm indicated that the strain had no drug resistance to antibiotics, the colony diameter of 9-17 mm had weak drug resistance, and the colony diameter larger than 17 mm had drug resistance (Xia et al., 2019).

### Optimization of culture conditions of antagonistic bacteria

The antagonistic bacteria was cultured in NA liquid medium and incubated in the dark at 28°C and pH 7.0 with shaking at 180 rpm. The OD_600_ value was measured every 4 h until 36 h, and then measured every 12 h until 84 h, NA liquid medium alone was used as a control. At each time point, OD_600_ was measured by a spectrophotometer at an absorbance wavelength of 600 nm (Zhang et al., 2019).

The pH was adjusted to 3.0, 5.0, 6.0, 7.0, 7.4, 8.0, 10.0, and 12.0 with NaOH and HCl, respectively, and 0.1 mL of bacterial suspensions was cultured in 100 mL of NA liquid medium with different pH values, shaken at 28°C, 180 r/min, and the OD_600_ value was measured at 600 nm after 48 h. NA liquid medium alone was used as a control, and three independent biological replicates were set for each treatment.

The optimum growth temperature curve was drawn with 0.1 mL of bacterial suspensions in 100 mL of NA culture medium (pH 7.4). The endophyte bacterial suspensions was cultured at different temperatures (4°C,10°C, 16°C, 22°C, 28°C, 34°C, 40°C, 46°C, and 52°C). The OD_600_ value was measured at 600 nm after 48 h. NA liquid medium alone was used as a control, and three independent biological replicates were set for each treatment.

A liquid medium with glucose (2.5 g/L) and peptone (10 g/L) as carbon source and nitrogen source, respectively, was used as the basic medium. Then, the carbon source of glucose was replaced by sucrose and lactose, and the nitrogen source of peptone was replaced by beef extract or ammonium nitrate to explore the best combination of carbon and nitrogen sources for endophyte fermentation.

### Effect of culture supernatant on mycelial morphology

0.5 mL of bacterial suspensions was inoculated into 50 mL of the optimized liquid medium and incubated for 2 days at 28°C with shaking at 180 r/min. The culture supernatant was collected from the culture broth by centrifugation at 8,000 rpm for 30 min at 4°C. The bacteria XY-1 in the culture supernatant was filtered out through a 0.22 µm microfiltration membrane. One group was poured 5 mL of the culture broth into a petri dish containing 10 mL of PDA medium, and inoculated the center of the dish with *F*. *graminearum* mycelia plug. The other group was inoculated with *F. graminearum* mycelia plug alone. Then they were cultured in a constant temperature incubator at 28°C for 3 to 5 days. The medium at the junction of the inhibition ring was observed under a microscope, and the morphological characteristics of the mycelium of the treatment group and the control group were compared. Each treatment contains 3 replicates.

### Wheat spikes inoculation antagonism tests

Testing for the antagonistic effect of endophyte against *F. graminearum* was performed on detached spikelets from wheat cultivar “Emai 170”. Detached spikelets were soaked in a suspension of endophyte or control water for 2 min, before being placed on plates filled with water agar medium (3 g/L). Five days later, a conidia suspension of *Fusarium graminearum* (10^5^ conidia/mL) was sprayed on spikelets. Under conditions of 20/15°C day/night temperature, 16/8 h light/dark photoperiod, and 65% of relative humidity. Two weeks later, morbidity, disease index and 1000-grain weight was recorded. Each treatment consisted of two independent experimental replicates with at least 30 spikelets per replicate.

### Statistical analysis

Data were subjected to analysis of variance using SPSS 19.0, where F tests were significant at p < 0.05, and the means were separated by Duncan’s Multiple Range test at p < 0.05.

## Results

### Selection of endophytic bacteria

**Figure 1:**
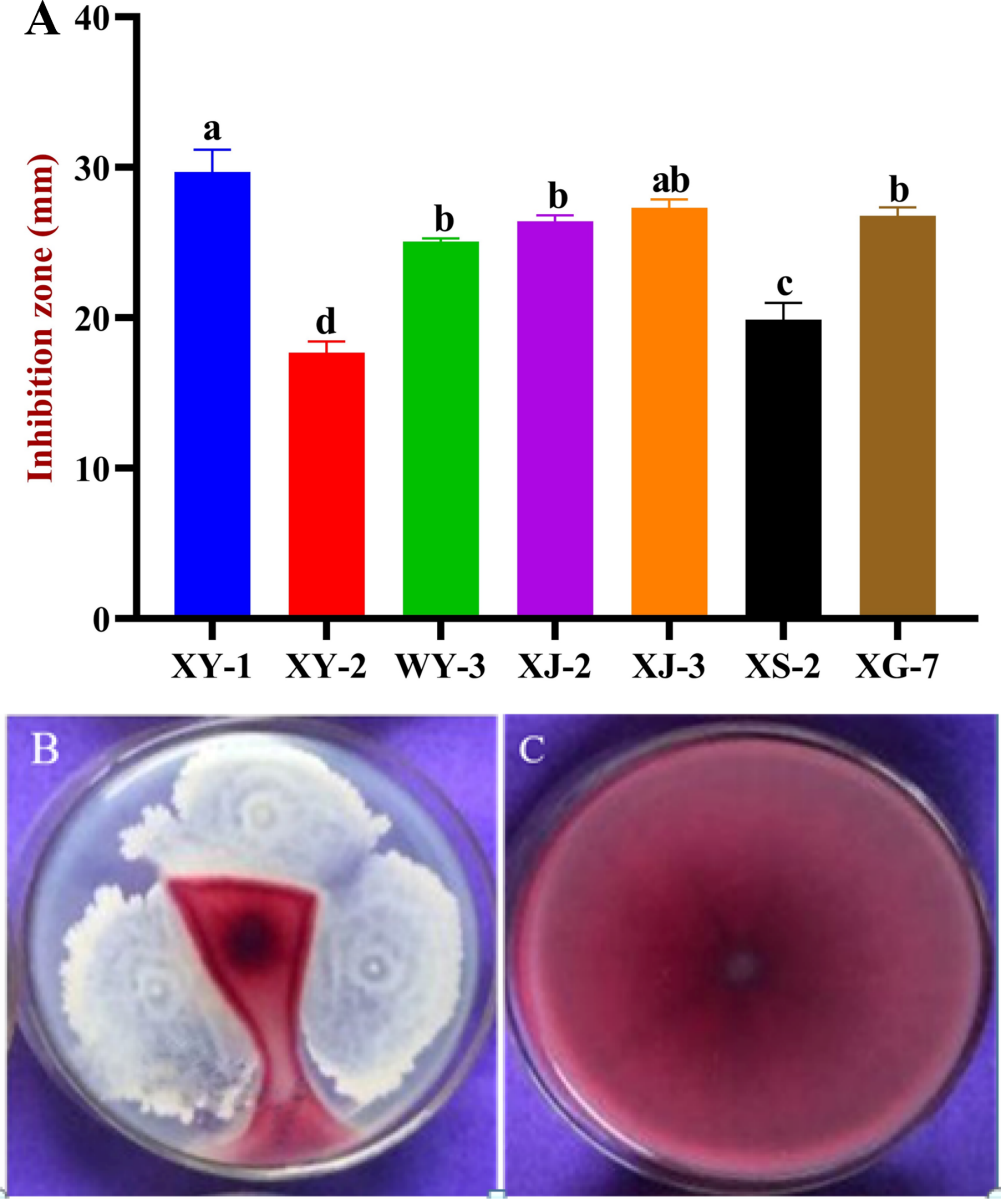
Antagonistic effects of endophytic bacteria on *Fusarium graminearum*. **(A)** Inhibition effects of six endophyte bacteria against *F. graminearum*. **(B)** The antagonistic effect of XY-1 against *F. graminearum*. **(C)** *F. graminearum* growth alone. Values are means ± SD (n = 10). Different letters above the bars indicate a significant difference at *p* < 0.05.

According to the differences of colony morphology and color, a total of 45 bacterial strains were isolated from different tissues of wheat. Seven strains antagonistic to *Fusarium graminearum* were screened by dual culture assays. Among them, the stain XY-1 isolated from root, exhibited the best antagonistic effect. The average diameter of the inhibition zone was larger than other strains, which was 29.7 mm (**Figure 1**). Therefore, XY-1 was selected for further study.

### Anti-fungal activity of strain XY-1

Strain XY-1 was tested for antagonism against several common crops and fruit tree pathogens by using dual culturing assays. The results showed that XY-1 displayed an inhibitory effect on the growth of four tested pathogens, namely *Colletotrichum gloeosporioides*, *Rhizoctonia solani, Sclerotium rolfsii*, and *Alternaria alternate.* The zone of inhibition ranged from 17.7 to 32 mm. In addition, XY-1 exhibited the best inhibitory effect on *Alternaria alternata*, followed by *Sclerotium rolfsii* (**Table 1**).

**Table 1:** Antagonistic activities of strain XY-1 against other four plant fungal pathogens.

Pathogens	Average inhibition zone diameter (mm)
*Colletotrichum gloeosporioides*	22.10 ± 3.11a
*Sclerotium rolfsii*	29.91 ± 0.90b
*Rhizoctonia solani*	19.80 ± 0.70c
*Alternaria alternata*	30.00 ± 1.39d

Data are means ± SD (n = 3). Different letters indicate a significant difference among treatments at p < 0.05 level. The data were subjected to one-way ANOVA with Duncan's multiple range test using SPSS 19.0 software.

### The optimal fermentation conditions of strain XY-1

**Figure 2:**
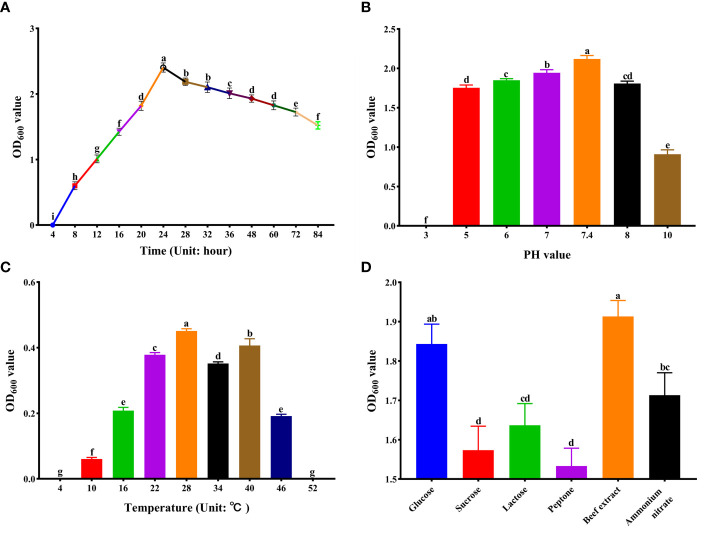
Optimization of Culture Conditions for strain XY-1. **(A)** Growth curve. **(B)** Growth in different pH. **(C)** Growth in different temperatures. **(D)** Growth in different carbon and nitrogen sources. Values are means ± SD (n = 6). Different letters above the bars indicate a significant difference at *p* < 0.05.

In order to observe the growth of strain XY-1, the bacterial solution was inoculated into the NA liquid medium, incubated at 28°C with shaking (160 r/min), and its OD_600_ value was measured at a wavelength of 600 nm. As shown in **Figure 2A**, XY-1 was in the exponential phase within 24 h after inoculation into the NA culture medium; it was in the stable phase within 24-60 h of inoculation; it entered the recession phase after 60 h (**Figure 2A**).

**Figure 2B** showed that XY-1 could grow at pH 4.0-10.0, but not at pH 3.0. The pH range for growth was 5.0 to 9.0, with an optimum pH of 7.4 (**Figure 2B**). Strain XY-1 was placed in NA broth at pH 7.4 and placed at different temperatures. The results showed that strain XY-1 could grow at 10–50°C with an optimum temperature of 28°C (**Figure 2C**).

In this experiment, sucrose, glucose and lactose were used as carbon sources, and peptone, beef extract and ammonium nitrate were used as nitrogen sources. The results showed that XY-1 could utilize all carbon and nitrogen sources tested in this study. Among them, glucose was the most suitable carbon source, and beef extract was the most suitable nitrogen source (**Figure 2D**).

### Antibiotics tolerance cultivation

In order to test the resistance of the strain XY-1 to common antibiotics, *chloramphenicol* (Ch1), *streptomycin* (Str), *tetracycline* (Tet), *kanamycin* (Kan), and *ampicillin* (Amp) was placed in NA liquid medium, respectively. The plaque diameters were counted after culturing in a constant temperature incubator at 28°C for 2 days. The results showed that the resistance of XY-1 to the five antibiotics was different, that XY-1 had better resistance to *chloramphenicol* and *ampicillin* (**Table 2**).

**Table 2:** Antagonistic bacteria XY-1 drug resistance experimental results.

Antibiotic	Concentration (mg/mL)	Average colony diameter(mm)	Tolerance
*Chloramphenicol* (Ch1)	25	25.00	Strong effect
*Ampicillin* (Amp)	100	25.30	Strong effect
*Tetracycline* (Tet)	25	16.80	weak effect
*Streptomycin* (Str)	50	8.00	no effect
*Kanamycin* (Kan)	10	8.00	no effect
CK	---	43.00	-----

### Identification of strain XY-1

**Figure 3:**
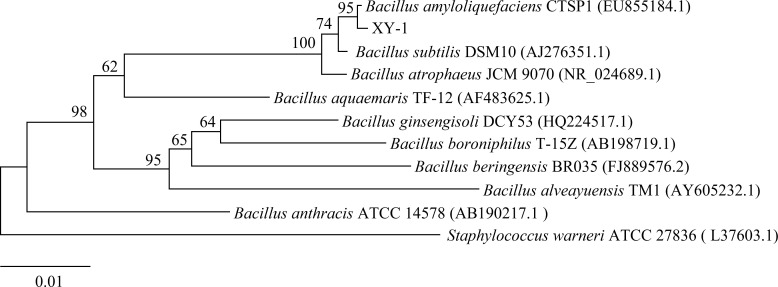
Phylogenetic trees of strain XY-1 based on 16S rDNA sequence. The phylogenetic tree was constructed using MEGA7.0 software with the maximum likelihood method. Bar indicates the numbers of nucleotide substitutions per site.

The sequencing data showed that the length of the 16S rDNA sequence of the strain XY-1 was 1207 bp. The sequence was blast aligned in the NCBI database (http://www.ncbi.nlm.nih.gov/), and the phylogenetic tree was constructed using Mega7.0 software (**Figure 3**). The analysis showed that XY-1 belonged to *Bacillus*, and the sequence similarity with *Bacillus amyloliquefaciens* (EU855184.1) was 95%. Based on its physiological and biochemical characteristics, the strain XY-1 was preliminarily classified as *Bacillus amyloliquefaciens*.

### Culture supernatant affects *F. graminearum* growth

**Figure 4:**
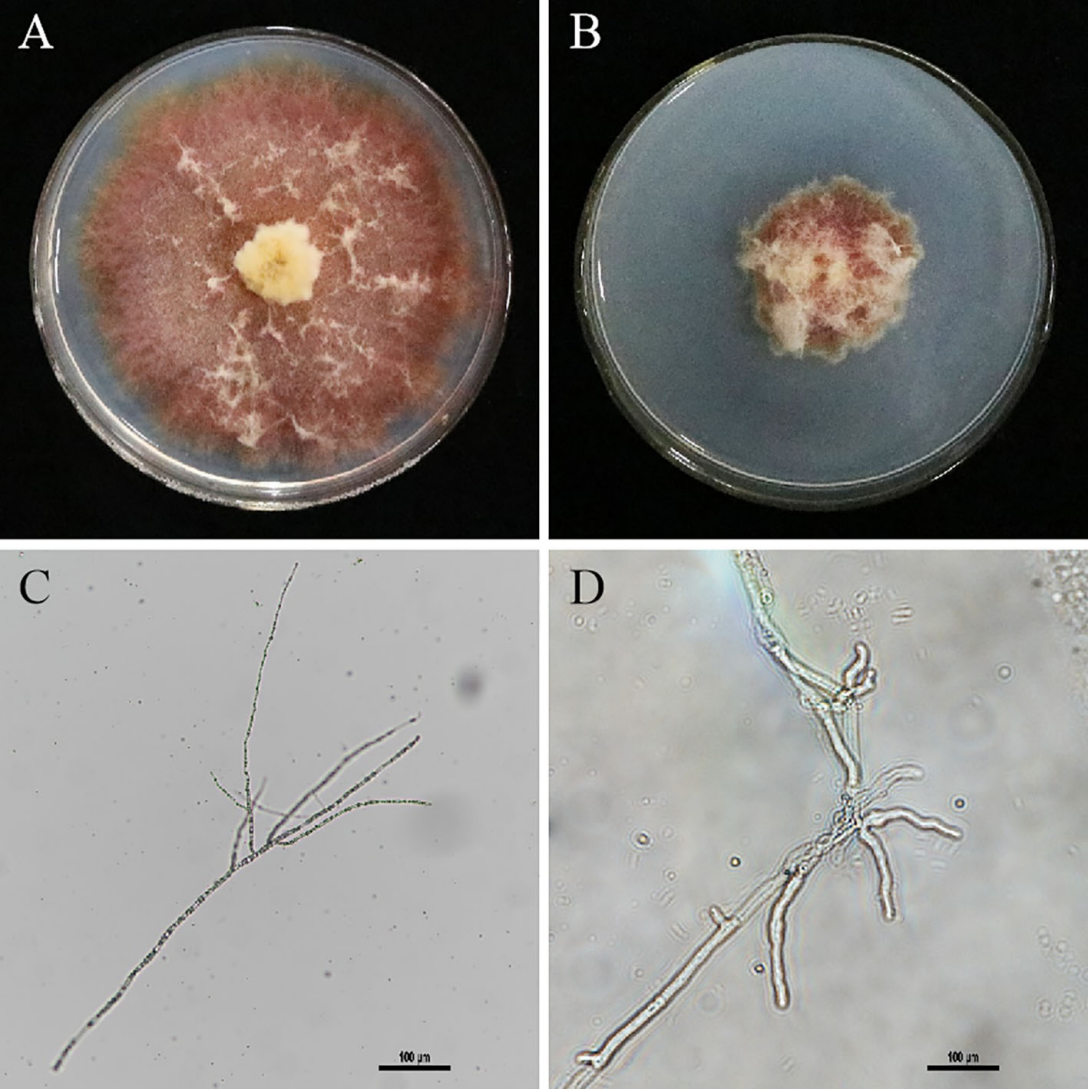
Effect of culture broth on mycelium morphology. **(A)** *F. graminearum* growth alone. **(B)** The antagonistic effect culture supernatant against *F. graminearum*. **(C)** The mycelium morphology of *F. graminearum* grown alone under microscope. **(D)** The mycelium morphology of Figure B with culture supernatant under microscope.

The medium at the junction of the inhibition ring was observed under a microscope, and the morphological characteristics of the mycelium of the treatment group and the control group were compared. The results showed that the hyphae of *F. graminearum* grew alone normally (**Figure 4A**), and the hyphae of the pathogenic fungi with culture supernatant of XY-1 were obviously inhibited (**Figure 4B**). As shown in **Figure 4C**, the mycelium morphology of *F. graminearum* grown alone was normal, slender and long (**Figure 4C**). By comparison, the mycelium morphology of *F. graminearum* with culture supernatant was malformation, thick and short (**Figure 4D**).

### The antagonistic effect of endophyte on *F. graminearum*

**Figure 5:**
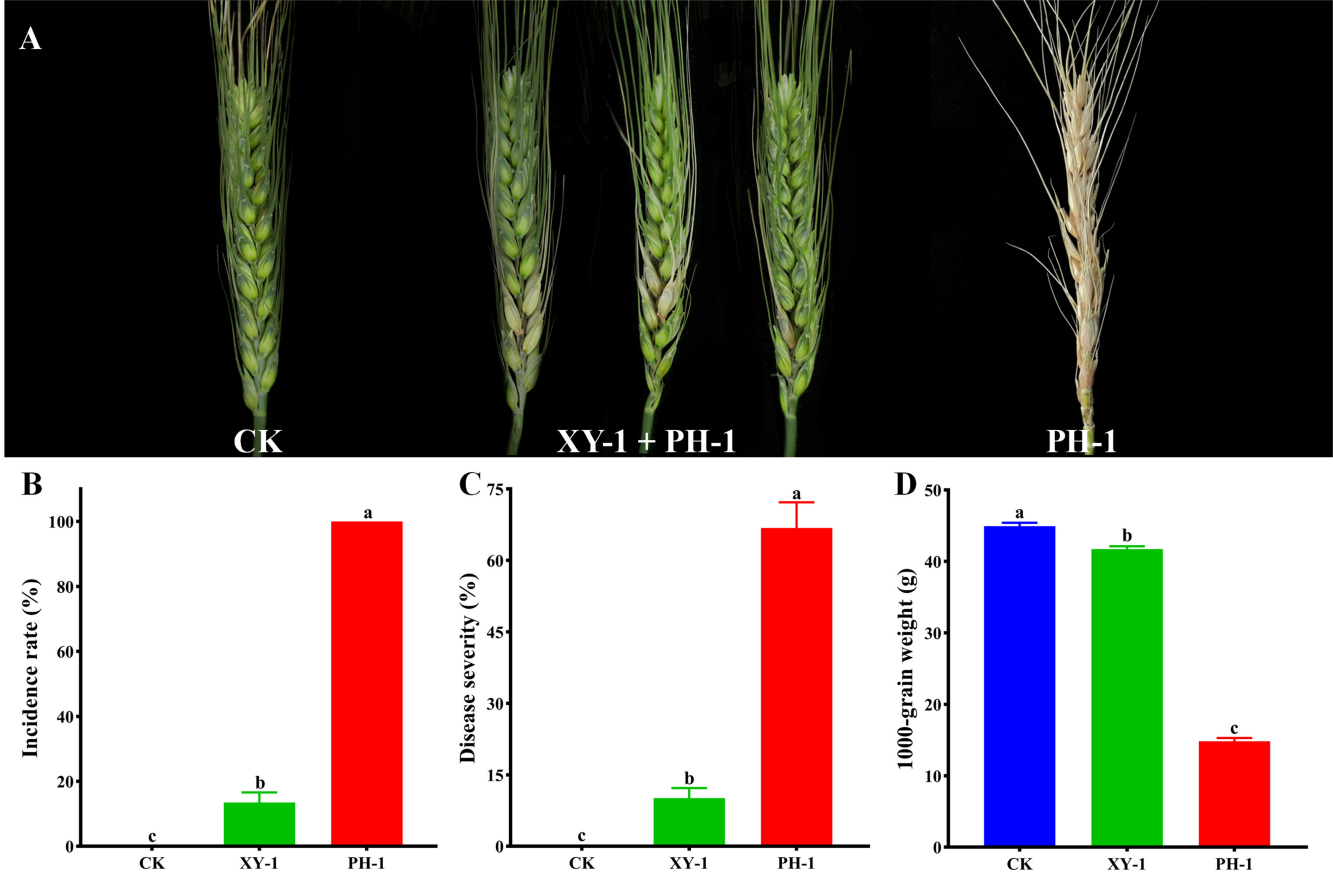
Antagonistic of XY-1 against *F. graminearum* on spikes. **(A)** Distilled water was used as a CK, symptoms of wheat heads inoculated with XY-1 plus PH-1 and FHB alone; **(B)** Incidence of wheat scab of CK, XY-1 plus PH-1 and PH-1 alone; **(C)** Disease index of CK, XY-1 plus PH-1 and PH-1 alone; **(D)** 1000-grain weight of CK, XY-1 plus PH-1 and PH-1 alone. Incidence is calculated using the following formula: n/N × 100%, where n represents the number of diseased wheat spikes, respectively, and N represents the total number of wheat spikes used. Disease index is calculated using the following formula: (n0 × 0 + n1 × 1 + n2 × 2 + n3 × 3)/3N × 100%, where n0, n1, n2, and n3 represent the number of wheat heads exhibiting disease levels 0, 1, 2, and 3, respectively, and N represents the total number of wheat spikes used. Values are means ± SD (n = 6). Different letters above the bars indicate a significant difference at p < 0.05.

The antagonistic effect of XY-1 against *F. graminearum* was further tested in isolated wheat spikelets. Spikes inoculated with alone developed typical wheat head blight symptoms within two weeks, and inoculated with distilled water alone did not cause any visible disease symptom (**Figure 5A**). As shown in **Figure 5B**, CK were the isolated spikelets that were not inoculated with *Fusarium graminearum*, and they were symptomless. The incidence rate of isolated spikelets that were immersed in the filtered culture supernatant and then inoculated with PH-1 was 13.5%, and the incidence rate of the isolated spikelets that were inoculated with *F. graminearum* alone was 100%. The results showed that the culture supernatant of strain XY-1 reduced the incidence of *F*. *graminearum* by 86.5% (**Figure 5B**). The study showed that bacteria XY-1 reduced the disease index by 56.7% (**Figure 5C**). The 1000-grain weight measurement data for each group of wheat showed that the group inoculated with *F. graminearum* alone was 15 g, the treatment group with culture supernatant of XY-1 just reduced by 3g compared with CK (**Figure 5D**). The application of XY-1 greatly reduced the yield decline caused by FHB.

## Discussion

The rapid occurrence and prevalence of FHB in warm and humid environments not only causes serious economic losses, but also causes food safety problems. Therefore, FHB has become the important limiting factors threatening the safe production of wheat. However, biological control of wheat and other crops in the early stage can reduce the outbreak of head blight and protect the yield and quality of crops (Li et al., 2007; Wen et al., 2012). In this study, experimental strains were collected from the occurrence area of FHB. And a strain XY-1 which was identified as *Bacillus amyloliquefaciens*, had a highly antagonistic effect on *F. graminearum*.

*Bacillus amyloliquefaciens* has strong stress resistance, and its secondary metabolites have different degrees of inhibitory effect on all kinds of pathogens. It is one of the main strains in biocontrol research at present. *B. amyloliquefaciens* can induce host defense responses and increase the enzymatic activity associated with disease resistance. The content of growth-promoting hormones such as gibberellin and auxin in plants inoculated with *B. amyloliquefaciens* was higher, and the activities of defense-related enzymes such as peroxidase were also higher (Zhang et al., 2020). *B. amyloliquefaciens* TWC2 can induce the activity of resistance enzymes in sugarcane and enhance the disease resistance of plants (Liang et al., 2018). *B. amyloliquefaciens* can produce volatile substances during the growth process to inhibit the growth and development of pathogenic fungi. The 34 volatile substances produced by *B. amyloliquefaciens* LJ02 have inhibitory effects on various pathogenic fungi such as Black rot fungus (Hao et al., 2016). Among the 36 volatile substances produced by *B. amyloliquefaciens* NJN-6, 11 kinds of gases can completely inhibit the growth of *Fusarium oxysporum* and have a preventive effect on banana Fusarium wilt (Yuan et al., 2012).

Through the determination of physiological and biochemical characteristics of strain XY-1. *B. amyloliquefaciens* XY-1 not only had highly antagonistic effect on FHB, but also had good resistance to four fungal pathogens, *Colletotrichum gloeosporioides*, *Rhizoctonia solani*, *Sclerotium rolfsii*, and *Alternaria alternata*. So XY-1 could be used as a biological control resource in the prevention and control of various diseases. At the same time, XY-1 showed strong resistance to *chloramphenicol* and *ampicillin*, which further highlights the application potential of the strain in biological control.

Winter Wheat Region in the Middle and Lower Reaches of the Yangtze River, China, FHB is one of the main diseases affecting wheat production (Ma and Lu, 2010). For many years, spraying carbendazim has been the main measure to control FHB in production. But carbendazim can induce drug resistance in fungal pathogens and promote the synthesis of DON toxin, resulting in more serious and wider spread of FHB in China (Li et al., 2006). The key to biological control is to inhibit the occurrence of field diseases, so we will require large-scale and systematic field trials in different pilots and different years to verify the actual biological control effect of the strains. The active ingredients will be screened from the culture supernatant of the antagonist XY-1. The compound with better antifungal effect will be isolated and its antifungal mechanism will be explored. This study will provide materials for the development of bacterial agents for biological control of FHB.

## Conclusion

In this study, the strain XY-1 with the best antagonistic effect on FHB was screened out of 7 endophytes isolated from wheat roots using dual culture assays. The 16S rDNA sequence identified that the antagonistic XY-1 was *Bacillus amyloliquefaciens*. Further research found that XY-1 also had antagonistic activity against *Colletotrichum gloeosporioides*, *Rhizoctonia solani*, *Sclerotium rolfsii*, and *Alternaria alternata*. Antibiotics tolerance cultivation showed that XY-1 had strong resistance to *Chloramphenicol* and *Ampicillin.* The strain XY-1 displayed strong antifungal activity against *F. graminearum*. In conclusion, this study provides a novel *B. amyloliquefaciens* strain which is a powerful biocontrol bacterium.

## Data availability statement

The original contributions presented in the study are included in the article/supplementary material. Further inquiries can be directed to the corresponding authors.

## Author contributions

DM and HS designed this article; XX and YC directed the data analysis and manuscript writing. DM and JY supervised the experiment. ZF confirmed the manuscript. All authors contributed to the article and agreed to submit version of the manuscript.

## Funding

This research was supported by “Open Project Program of Engineering Research Center of Ecology and Agricultural Use of Wetland, Ministry of Education (KFT202102)”, and “the National Key Research and Development Program of Jiangsu (BE2021335)”.

## Conflict of interest

The authors declare that the research was conducted in the absence of any commercial or financial relationships that could be construed as a potential conflict of interest.

## Publisher’s note

All claims expressed in this article are solely those of the authors and do not necessarily represent those of their affiliated organizations, or those of the publisher, the editors and the reviewers. Any product that may be evaluated in this article, or claim that may be made by its manufacturer, is not guaranteed or endorsed by the publisher.
